# Risk factors for domestic physical violence: national cross-sectional household surveys in eight southern African countries

**DOI:** 10.1186/1472-6874-7-11

**Published:** 2007-07-16

**Authors:** Neil Andersson, Ari Ho-Foster, Steve Mitchell, Esca Scheepers, Sue Goldstein

**Affiliations:** 1 Centro de Investigación de Enfermedades Tropicales (CIET), Universidad Autónoma de Guerrero, Acapulco, México; 2 CIET Trust, 71 Oxford Road, Saxonwold 2193, South Africa; 3 Soul City Institute for Health and Development Communication, 2nd Floor Park Terrace, Parktown, Johannesburg, South Africa

## Abstract

**Background:**

The baseline to assess impact of a mass education-entertainment programme offered an opportunity to identify risk factors for domestic physical violence.

**Methods:**

In 2002, cross-sectional household surveys in a stratified urban/rural last-stage random sample of enumeration areas, based on latest national census in Botswana, Lesotho, Malawi, Mozambique, Namibia, Swaziland, Zambia and Zimbabwe. Working door to door, interviewers contacted all adults aged 16–60 years present on the day of the visit, without sub-sampling. 20,639 adults were interviewed. The questionnaire in 29 languages measured domestic physical violence by the question "In the last year, have you and your partner had violent arguments where your partner beat, kicked or slapped you?" There was no measure of severity or frequency of physical violence.

**Results:**

14% of men (weighted based on 1,294/8,113) and 18% of women  (weighted based on 2,032/11,063) reported being a victim of partner physical violence in the last year. There was no convincing association with age, income, education, household size and remunerated occupation. Having multiple partners was strongly associated with partner physical violence. Other associations included the income gap within households, negative attitudes about sexuality (for example, men have the right to sex with their girlfriends if they buy them gifts) and negative attitudes about sexual violence (for example, forcing your partner to have sex is not rape). Particularly among men, experience of partner physical violence was associated with potentially dangerous attitudes to HIV infection.

**Conclusion:**

Having multiple partners was the most consistent risk factor for domestic physical violence across all countries. This could be relevant to domestic violence prevention strategies.

## Background

Domestic violence – also known as intimate partner abuse, family violence, wife beating, battering, marital abuse, and partner abuse – is an international problem[1,2]. Domestic violence is not a single behaviour but a mix of assaulting and coercive physical, sexual, and psychological behaviours designed to manipulate and dominate the partner to achieve compliance and dependence. Women are more likely to experience physical injuries or psychological consequences[3,4].

Domestic violence is well documented in several African countries. In eastern Nigeria, a clinic-based survey of 300 women reported 40% had experienced violence in the previous year[5]. In one district of Uganda, 30% of 5,109 women attending a clinic had received threats or physical abuse. The majority of respondents viewed wife beating as justifiable in some circumstances[6]. In Durban, South Africa, more than one third of women from a low-income community had experienced domestic violence at some stage[7]. A South African study reported domestic violence associated with violence in childhood, education and multiple partners[8,9]. In southern Africa domestic violence is particularly important because of the multiple links between violence and HIV infection[10]. Links between domestic violence and HIV have been reported in Botswana[11], Ghana[12], Malawi[13], South Africa[14], Tanzania[15], Uganda[16,17], Democratic Republic of Congo[18] and Zambia[19].

This is a baseline assessment of attitudes and practices, from which we intend to measure the impact of mass media campaigns, launched since the baseline by Soul City. The survey content was thus geared to measure the impact of education-entertainment messages[20], rather than as a specific research hypothesis. One section of the questionnaire dealt with domestic violence – attitudes and subjective norms, collective efficacy, discussion of the issue and experience of physical domestic violence in the last year – and the results are reported here as a cross-sectional survey.

## Methods

### Design

In Botswana, Lesotho, Swaziland, Malawi, Mozambique, Namibia, Zambia and Zimbabwe we stratified the most recent available census into rural, urban (not within the capital region), and urban capital sites. In each country, we drew a last stage random selection of enumeration areas, with probability proportional to the national population (Table 1).

**Table 1:** Sample weights in each country

	Botswana	Lesotho	Malawi	Mozambique	Namibia	Swaziland	Zambia	Zimbabwe	TOTAL
Sample population	13689	16812	34488	9030	9898	14512	16189	12346	126964
% rural (sample population)	45%	83%	85.1%	51.6%	58.3%	74.5%	65.6%	63.6%	70.2%
Rural weight (Actual pop/sample pop)	1.016	0.971	1.005	1.316	1.032	1.033	1.046	1.047	1.007
% urban (sample population)	47%	4.7%	3.8%	37.2%	26.6%	18.5%	21.4%	17.3%	18.0%
Urban weight (Actual pop/sample pop)	0.923	2.034	1.304	0.693	0.892	0.909	0.835	1.003	1.019
% capital (sample population)	8.1%	12.2%	11.1%	11.2%	15.1%	7%	13.1%	19.2%	11.8%
Capital weight (Actual pop/sample pop)	1.356	0.799	0.855	0.564	1.067	0.890	1.039	0.840	0.927
% country (sample population)	10.8%	13.2%	27.2%	7.1%	7.8%	11.4%	12.8%	9.7%	100%
Country weight(Actual pop/sample pop)	0.298	0.254	0.708	4.160	0.407	0.158	1.343	2.315	1.000

### Training and fieldwork

After training, coordinators translated, back-translated and piloted the common instruments in 29 languages: Afrikaans, Bemba, Changana, Chichewa, Chindali, Chitimbuka, Chona/Shona, Chope, English, Herero, Kalanga, Kaonde, Kwangali, Lozi, Luvale, Mucua, Ndau, Ndebele, Nyanja, Oshiwambo, Portuguese, Ronga, Sena, Sesotho, Seswati, Setswana, Shangaan, Xitshwa and Xitsonga. Each field team of seven or eight interviewers visited approximately 10 communities, one per day. Interviewers tried to cover all households in each enumeration area, without sub-sampling. In each household, they interviewed all adults aged 16–60 years present at the time of the visit.

### Ethical considerations

An accredited international ethical review board evaluated the proposal, noting concerns that disclosure might place the respondent at risk and that the questions about sexuality probed confidential issues. Interviewers informed each respondent of their right to refuse to participate, and of their right to refuse to answer any question. Before starting the questionnaire, the interviewers requested verbal consent to proceed. They did not record names or other identifying feature, and took precautions that the interview was out of hearing of others.

### Participants

Of the 17,377 households in 213 randomly selected enumeration areas, 20,639 adults participated from 16,707 households (96% initial acceptance) where 85,114 people lived. 58% (11,872/20,639) were female; 63% (13,017) were rural  residents, 22.1% (4,563) urban and 14.8% (3,059) lived in the capital/metro  area (Table 2).

**Table 2:** Characteristics of the sample population

		Botswana	Lesotho	Malawi	Mozambique	Namibia	Swaziland	Zambia	Zimbabwe	TOTAL
Number of adults interviewed	Adults	2526	2367	2863	2458	2649	1974	2963	2842	20639
% who had not completed primary school	Crude	322/2367	639/2183	1572/2827	1807/2425	497/2599	401/1827	803/2895	207/2772	6248/19895
	Weighted	12%	22%	43%	75%	17%	14%	22%	6%	42%
% female respondents	Crude	1495/2489	1488/2348	1683/2853	1471/2446	1465/2632	1122/1957	1605/2954	1543/2827	11872/20506
	Weighted	57%	66%	63%	61%	56%	56%	58%	54%	59%
% who said they did not have enough food in the last week	Crude	616/2216	734/2020	869/2180	705/1752	537/1799	672/1816	821/2175	815/2616	5769/16574
	Weighted	27%	31%	27%	42%	27%	23%	36%	29%	35%
% with no income	Crude	248/1900	419/1963	51/1983	66/1628	302/1727	230/1584	132/1890	155/2067	1603/14742
	Weighted	11%	17%	2%	4%	14%	8%	5%	6%	5%
Average HH size	Average	4.6	4.7	4.8	4.9	5.4	6.2	3.9	4.7	5.3

### Outcome measures

We defined domestic physical violence by responses to the question: "In the last year, have you and your partner had violent arguments where your partner beat, kicked or slapped you?" To facilitate disclosure, interviewers asked this with the respondent alone. If this was not possible, they noted presence of a listener. Interviewers read questions without additional explanations, and recorded answers verbatim. Wherever possible, female researchers interviewed women and male researchers interviewed men. With the exception of one question about pregnancy, interviewers administered the same instrument to men and women.

We limited domestic violence to reports of physical abuse, and we had no measure of severity of the violence. We included items on attitudes to and subjective norms of domestic violence, collective efficacy to reduce domestic violence (Can your community do anything about violence against women?) and discussion of domestic violence (In the last year, how often did you talk with anyone about domestic violence? To whom did you speak most often about domestic violence?). In designing the evaluation of the impact of mass media, we anticipated that some effect might be measured in these intermediate outcomes before changing the actual occurrence of domestic physical violence.

The relevance of partner physical violence to HIV/AIDS risk came from answers to the questions "Do you think you are at risk of getting HIV?" and "If you found you were HIV positive, how would you change your sex life", considering "always use a condom" and "abstain from sex" as positive values. Negative values included "no change", "spread it intentionally", "same partner" and "sleep with virgin to cure".

### Analysis

Data technicians manually digitised questionnaire data twice and eliminated keystroke errors by verifying discordant entries with the original questionnaires. We weighted final estimates in line with the national populations and the eight-country estimates weighted national indicators by the population of each country (Table 1). In a univariate analysis, we stratified each association between partner physical violence and potential risk factors by each of the others in turn (List 1, see Appendix), initially ignoring multiple influences[21,22]. We adjusted for the multiple comparisons by requiring 99% confidence.

For risk factors not explained by any stratifying variable and those with multiple influences, a step down logistic regression model tested the effect of country, age, sex, education, income, food security, household size, occupation, and the factors in List 1 (see Appendix). The several items on attitudes to sexuality and violence showed co-linearity, with no single variable attaining statistical significance in the preliminary logistic regression model. We included the variable from each group that showed the strongest association with the outcome in the model.

## Results

Some 16% of men (weighted value based on 1,294/8,113) and 18%  of women (weighted value based on 2,032/11,063) reported partner physical  violence in the last year; 6.8% (809/11,872) of female respondents and 6.0%  (521/8,634) of males declined to answer this question. The lowest rates of  partner physical violence came from Mozambique (9%) and Malawi (9%) and the  highest from Zambia (32%) (Tables 3 and 4). The 7.1% with someone else present at the time of the interview were *more *likely to report a violent altercation (OR 1.18, 95%CI 1.02–1.35;  285/1,459 compared with 2,974/17,381 alone at the time).

**Table 3:** MALE Experience of physical violence in the last year (beat, kicked or slapped), discussion about gender violence and participation in community action about violence against women

		Botswana	Lesotho	Malawi	Mozambique	Namibia	Swaziland	Zambia	Zimbabwe	TOTAL
% (number) who had, in the last year, had violent arguments where a partner beat, kicked or slapped the respondent, of those who answered	Crude	189/929	91/768	72/1109	70/930	168/1113	162/1261	337/1261	205/1231	1294/8113
	Weighted	21%	12%	6%	8%	15%	21%	27%	17%	16%
	Missing	65	92	61	45	54	63	88	53	521
% who said they had not spoken with anyone about gender violence in the last year	Crude	638/960	489/825	748/1167	657/964	679/1152	515/798	803/1329	590/1271	5119/8466
	Weighted	66%	57%	64%	69%	59%	65%	60%	46%	60%
	Missing	34	35	3	11	15	37	20	13	168
% who had participated in community activities in the last year	Crude	71/930	47/785	44/1159	64/964	64/1142	29/772	48/1328	118/1242	485/8322
	Weighted	8%	6%	4%	6%	6%	4%	4%	9%	6%
	Missing	64	75	11	11	25	63	21	42	312
% (number) who consider violence against women a serious problem in their community	Crude	758/928	796/1152	796/1152	613/952	791/1134	505/773	722/1298	580/1220	5242/8257
	Weighted	82%	60%	69%	64%	70%	65%	56%	47%	64%
	Missing	66	60	18	23	33	62	51	64	377
% (number) who said their community CAN do anything about violence against women	Crude	692/899	479/767	663/1150	508/903	626/1108	434/732	545/1255	582/1014	4529/7828
	Weighted	77%	64%	58%	56%	56%	59%	43%	57%	58%
	Missing	95	93	20	72	59	103	94	270	806

**Table 4:** FEMALE Experience of physical violence in the last year (beat, kicked or slapped), discussion about gender violence and participation in community action about violence against women

		Botswana	Lesotho	Malawi	Mozambique	Namibia	Swaziland	Zambia	Zimbabwe	TOTAL
% (number) who had, in the last year, had violent arguments where a partner beat, kicked or slapped the respondent, of those who answered	Crude	257/1371	207/1309	176/1586	148/1374	233/1382	221/1034	538/1509	252/1498	2032/11063
	Weighted	19%	16%	11%	11%	17%	21%	36%	17%	19%
	Missing	124	179	97	97	83	88	96	45	809
% who said they had not spoken with anyone about gender violence in the last year	Crude	1011/1424	741/1433	1203/1671	1009/1458	795/1452	648/1076	948/1586	722/1523	7077/11623
	Weighted	71%	52%	72%	70%	55%	60%	60%	48%	61%
	Missing	71	55	12	13	13	46	19	20	249
% who had participated in community activities in the last year	Crude	99/1401	53/1388	29/1659	76/1451	67/1425	29/1051	41/1576	142/1507	536/11458
	Weighted	7%	4%	2%	5%	5%	3%	3%	9%	5%
	Missing	94	100	24	20	40	71	29	36	414
% (number) who consider violence against women a serious problem in their community	Crude	1110/1364	856/1393	1164/1659	872/1421	1034/1420	699/1027	934/1523	777/1451	7446/11257
	Weighted	81%	62%	70%	59%	73%	68%	61%	53%	66%
	Missing	131	95	24	51	45	95	82	92	615
% (number) who said their community CAN do anything about violence against women	Crude	1002/1339	479/767	663/1150	508/903	626/1108	434/732	545/1255	582/1014	4529/7828
	Weighted	75%	63%	45%	50%	58%	55%	45%	52%	55%
	Missing	156	172	34	123	59	182	136	325	1187

### Personal and household factors

#### Sex

The gender gap in reported domestic physical was negligible in Botswana, Lesotho, Namibia, Swaziland and Zimbabwe. Elsewhere, female respondents reported being the subjects of partner physical violence more frequently than did male respondents: in Malawi, the population weighted rates were 7% and 11% for  males and females respectively (based on 72/1,109 and 176/1,586); in  Mozambique, 7% and 11% respectively (based on 70/930 and 148/1,374) and in  Zambia, 27% and 36% (based on 337/1,261 and 538/1,509).

#### Age

Respondents aged 30–39 years reported violent altercations  more  commonly (20.4% unweighted, based on 908/4,478), with lower rates among  older and younger respondents (16–19 years 11.4% 365/3,211; 20–29 years  19.3% 1,518/7,931; 40–49 years, 17.3% 376/2,196; 50–59 years 12.1%  135/1,118; and 60–66 years, 11.0% 26/235).

#### Home language

We found high reported rates of domestic physical violence in four of 29 interview languages. No less than 54% (82/152) of Lozi speakers (Zambia) reported partner physical violence in the last year. From the same country, 46% (99/197) of Tonga, 34% (339/995) of Bemba and 28% (206/744) Nyanja responders reported partner physical violence.

#### Education

Some 31% (6,248/19,895) of the respondents had completed  primary school; 3.5% (744/20,639) declined to answer this question. At  first  glance, the average person who had not completed primary school seemed more  likely to report partner physical violence: OR 1.18 99%CI 1.05–1.32  (2,350/12,016 among those who had not completed primary education compared  with 931/5,933 who had done so reported a violent altercation with a partner). This effect disappears entirely when stratifying by country; the levels of education combined with quite different rates of  violent altercation seem to confound the measurement. In Zambia, the only country where education was associated with violent altercations, the average person who had not completed primary school was *less *likely to report a violent argument with a partner: argument with a partner: OR 0.82 95%CI 0.69–0.98 (600/1,979) among those who had not completed primary education compared with 266/768 who had done so reported a violent altercation with a partner).

#### Household size

We could find no obvious trend of violent altercation with increasing household size; missing data 6.6% (1,360/20,639). The average person living in a household with more than five members was *less *likely to report a violent altercation than one living in a household of 1–5 people (OR 0.88 99%CI 0.63–0.98; 1,295/7,887 in higher occupancy households compared with 2,049/11,383 in lower occupancy households reported a violent altercation).

#### Urban/rural residence

Most respondents lived in rural areas (63.1% or 13,017/20,639); a further 22.1% were urban (4,563/20,639) and 14.8% lived in the capital city (3,059/20,639). There was very little difference in partner physical violence: rural 17.8% (2,164/12,160), urban 17.2% (736/4,287) and capital 15.8% (447/2,837).

#### Total household income

One in every ten (1,940/18,370) reported no income in the last  month (11% or 2,269/22,630 declined to answer this question). Stratifying  by  country, there was no convincing association of domestic physical violence  with income (OR adjusted 1.08, 99%CI 0.85–1.53; 346/1,757 of those with no  income and 27,017/15,458 of those with an income). There was no detectable  gender difference in this effect.

#### Remunerated occupation

One in every ten did not register an occupation (3.7%  751/20,639 missing data). Housewives were most likely to report partner  physical violence (25.6% based on 443/1,730), followed by those who  described themselves as unemployed (19.5% based on 812/4,169). There was  also no convincing association between remunerated occupation and partner  physical violence (OR 0.95, 99%CI 0.8–1.1). We constructed a new variable  to  reflect the "income gap" between personal employment and total household  income: overall, unemployed individuals in households with some income were  more likely to report domestic physical violence (OR 1.43 99%CI 1.27–1.60;  901/4,111 with the income gap and 2,091/12,722 without it reported physical  violence). On stratification by sex of respondent and country, however, it  turned out that this association is ascribed mostly to women in Namibia and  Zambia.

#### Food security

One in every three respondents reported having insufficient  food in the last week (34.5% unweighted, 7,070/20,475); 0.8% (164/20,639)  declined to respond. As with personal income, the average person reporting  insufficient food was slightly more likely to report partner physical  violence (OR 1.22 99%CI 1.10–1.35; 1,271/2,679 with insufficient food  reported, compared with 2,052/12,536 with sufficient food). We could not  explain this effect by urban/rural residence, country, attitudes to  sexuality or sexual violence or any the personal factors we documented.

### Attitudes about sexuality and sexual violence

Tables 5, 6, 7, 8, 9, 10 show the variation from country to country in attitudes about sexuality and sexual violence. Several of these beliefs were associated with partner physical violence (Tables 11 and 12): the belief that men have the right to have sex with girlfriends if they buy them presents (OR 1.42 99%CI 1.25–1.60), it is okay for an older man to have sex with teenagers (OR1.38 99%CI 1.20–1.59), women do not have the right to refuse sex with husbands and boyfriends (OR1.18 99%CI 1.05–1.30) and a person has to have sex to show love (OR 1.44 99%CI 1.38–1.59). Beliefs about gender violence were also associated with violent altercations: forcing one's partner to have sex is not rape (OR 1.23 99%CI 1.10–1.37) and women sometimes deserve to be beaten (OR1.56 99%CI 1.4–1.72). These associations were not explained by country, education, sex, remunerated occupation, income, multiple partners, household factors (like crowding, language, food security), or other attitudes and beliefs about sexuality or sexual violence.

**Table 5:** Male attitudes about sex

		Botswana	Lesotho	Malawi	Mozambique	Namibia	Swaziland	Zambia	Zimbabwe	TOTAL
% (number) who said women do not have the right to refuse to have sex with their husbands or boyfriends.	Crude	383/981	393/829	568/1165	538/970	436/1151	369/824	681/1255	614/1258	3982/8433
	Weighted	39%	47%	49%	55%	38%	45%	54%	49%	47%
	Missing	13	31	5	5	16	11	94	26	201
% (number) who said a person has to have sex with their boyfriend or girlfriend to show that they love them	Crude	350/983	528/838	505/1166	523/971	446/1152	407/821	596/1336	318/1277	3673/8544
	Weighted	36%	62%	44%	57%	39%	50%	45%	25%	44%
	Missing	11	22	4	5	15	14	13	7	90
% (number) who said it is okay for an older man to have sex with teenagers.	Crude	75/985	162/820	62/1168	196/972	111/1158	84/826	129/1343	105/1280	924/8552
	Weighted	8%	21%	5%	21%	10%	10%	10%	8%	11%
	Missing	9	40	2	3	9	9	6	4	82
% (number) who said men have the right to have sex with their girlfriends if they buy them gifts	Crude	172/980	331/822	285/1166	491/969	365/1154	189/827	509/1342	266/1280	2608/8540
	Weighted	18%	39%	25%	53%	32%	23%	38%	21%	31%
	Missing	14	38	4	6	13	8	7	4	94

**Table 6:** Female attitudes about sex

		Botswana	Lesotho	Malawi	Mozambique	Namibia	Swaziland	Zambia	Zimbabwe	TOTAL
% (number) who said women do not have the right to refuse to have sex with their husbands or boyfriends.	Crude	480/1466	594/1447	812/1679	772/1458	448/1457	429/1099	856/1516	662/1513	5053/11635
	Weighted	32%	40%	49%	52%	31%	39%	57%	44%	43%
	Missing	29	41	4	13	8	23	89	30	237
% (number) who said a person has to have sex with their boyfriend or girlfriend to show that they love them	Crude	428/1464	843/1452	763/1671	743/1461	411/1458	449/1104	651/1590	266/1533	4554/11733
	Weighted	29%	58%	46%	54%	28%	41%	42%	17%	39%
	Missing	31	36	12	10	7	18	15	10	139
% (number) who said it is okay for an older man to have sex with teenagers.	Crude	79/1470	226/1433	104/1679	289/1461	97/1459	108/1112	126/1596	134/1539	1163/11749
	Weighted	5%	16%	6%	20%	7%	10%	8%	9%	10%
	Missing	25	55	4	10	6	10	9	4	123
% (number) who said men have the right to have sex with their girlfriends if they buy them gifts	Crude	236/1468	534/1426	467/1671	651/1462	286/1450	186/1105	513/1593	216/1531	3089/11706
	Weighted	16%	37%	28%	48%	20%	17%	33%	14%	27%
	Missing	27	62	12	9	15	17	12	12	166

**Table 7:** Male attitudes about violence

		Botswana	Lesotho	Malawi	Mozambique	Namibia	Swaziland	Zambia	Zimbabwe	TOTAL
% (number) who said women sometimes deserve to be beaten	Crude	357/978	345/818	348/1166	395/968	505/1159	415/822	715/1337	421/1278	3501/8526
	Weighted	37%	41%	30%	41%	44%	51%	53%	33%	41%
	Missing	16	42	4	7	8	13	12	6	108
% (number) who said if a woman gets raped its her own fault	Crude	165/981	260/823	508/1162	452/970	209/1155	161/816	268/1337	178/1272	2201/8516
	Weighted	17%	31%	44%	49%	18%	20%	20%	14%	26%
	Missing	13	37	8	5	12	19	12	12	118
% (number) who said forcing sex with someone you know is not rape	Crude	242/982	302/824	299/1165	240/971	254/1158	84/821	346/1338	205/1281	1972/8540
	Weighted	25%	36%	26%	25%	22%	10%	26%	16%	23%
	Missing	12	36	5	4	9	14	11	3	94
% (number) who said Forcing your partner to have sex, is NOT rape	Crude	198/982	292/829	455/1166	309/971	401/1157	261/821	618/1340	395/1276	2929/8542
	Weighted	20%	35%	39%	33%	35%	32%	46%	31%	34%
	Missing	12	31	4	4	10	14	9	8	92
% (number) who said violence between a man and a woman is a private matter in which others shouldn't interfere	Crude	296/977	522/823	875/1165	546/970	497/1152	430/820	754/1335	628/1272	4548/8514
	Weighted	30%	63%	75%	58%	43%	53%	57%	50%	54%
	Missing	17	37	5	5	15	15	14	12	120

**Table 8:** Male attitudes about violence

		Botswana	Lesotho	Malawi	Mozambique	Namibia	Swaziland	Zambia	Zimbabwe	TOTAL
% (number) who said women sometimes deserve to be beaten	Crude	279/1459	426/1429	654/1677	539/1463	425/1454	436/1099	751/1592	368/1536	3878/11709
	Weighted	19%	30%	39%	38%	29%	40%	47%	24%	33%
	Missing	36	59	6	8	11	23	13	7	163
% (number) who said if a woman gets raped its her own fault	Crude	158/1463	339/1427	625/1673	544/1462	143/1458	120/1104	306/1591	171/1538	2406/11716
	Weighted	11%	24%	37%	39%	10%	11%	19%	11%	21%
	Missing	32	61	10	9	7	18	14	5	156
% (number) who said forcing sex with someone you know is not rape	Crude	324/1466	506/1428	437/1674	436/1462	259/1459	146/1108	448/1593	261/1535	2817/11725
	Weighted	22%	36%	26%	29%	18%	13%	28%	17%	24%
	Missing	29	60	9	9	6	14	12	8	147
% (number) who said Forcing your partner to have sex, is NOT rape	Crude	279/1467	509/1458	754/1676	536/1464	476/1457	371/1110	807/1592	515/1529	4247/11753
	Weighted	19%	35%	45%	36%	33%	34%	51%	34%	36%
	Missing	28	30	7	7	8	12	13	14	119
% (number) who said violence between a man and a woman is a private matter in which others shouldn't interfere	Crude	360/1458	809/1428	1335/1678	813/1461	556/1457	517/1102	831/1591	790/1523	6011/11698
	Weighted	24%	57%	80%	56%	38%	47%	52%	52%	51%
	Missing	37	60	5	10	8	20	14	20	174

**Table 9:** Male attitudes and subjective norms about sexual violence

% (number) who said		Botswana	Lesotho	Malawi	Mozambique	Namibia	Swaziland	Zambia	Zimbabwe	TOTAL
In my culture it is acceptable for a man to beat his wife	Crude	268/983	337/812	151/1163	317/965	327/1158	203/813	507/1329	382/1275	2492/8498
	Weighted	27%	41%	13%	33%	28%	25%	38%	30%	29%
	Missing	11	48	7	10	9	22	20	9	136
most people in our community feel women have a right to refuse sex with their partners	Crude	473/899	386/766	574/1142	461/957	741/1133	338/766	601/1251	505/1186	4079/8100
	Weighted	53%	52%	50%	49%	66%	44%	48%	43%	50%
	Missing	95	94	28	18	34	69	98	98	534
most people in our community feel forcing your partner to have sex is rape	Crude	650/943	509/777	616/1150	582/956	760/1138	491/801	602/1266	757/1212	4967/8243
	Weighted	69%	66%	54%	60%	67%	61%	47%	63%	60%
	Missing	51	83	20	19	29	34	83	72	391

**Table 10:** Female attitudes and subjective norms about sexual violence

% (number) who said		Botswana	Lesotho	Malawi	Mozambique	Namibia	Swaziland	Zambia	Zimbabwe	TOTAL
In my culture it is acceptable for a man to beat his wife	Crude	307/1449	495/1418	250/1674	441/1463	310/1451	183/1093	531/1587	425/1528	2942/11663
	Weighted	21%	35%	15%	32%	21%	17%	34%	28%	25%
	Missing	46	70	9	8	14	29	18	15	209
most people in our community feel women have a right to refuse sex with their partners	Crude	683/1317	682/1302	721/1647	675/1421	933/1423	528/1041	685/1444	685/1381	5592/10976
	Weighted	52%	54%	44%	49%	66%	50%	47%	50%	51%
	Missing	178	186	36	50	42	81	161	162	896
most people in our community feel forcing your partner to have sex is rape	Crude	912/1390	916/1351	793/1641	762/1423	926/1424	664/1064	673/1477	909/1440	6555/11210
	Weighted	66%	69%	48%	54%	65%	62%	45%	63%	59%
	Missing	105	137	42	48	41	58	128	103	662

**Table 11:** Male respondents: Associations with domestic physical violence (number of responses, Odds Ratio and 99%confidence interval)

		Botswana		Lesotho		Malawi		Mozambique		Namibia		Swaziland		Zambia		Zimbabwe		Overall	
		Partner violence		Partner violence		Partner violence		Partner violence		Partner violence		Partner violence		Partner violence		Partner violence		Partner violence	

		Yes	No	Yes	No	Yes	No	Yes	No	Yes	No	Yes	No	Yes	No	Yes	No	Yes	No

Reported having multiple partners	Yes	85	222	57	307	26	174	40	382	65	225	101	256	144	280	109	282	627	2128
	No	86	398	23	249	45	810	30	466	96	654	40	233	181	598	91	616	592	4024
	OR(99%CI)	**1.77 (1.13–2.77)**		**2.01 (1.03–3.90)**		**2.69 (1.41–5.14)**		1.63 (0.86–3.09)		**1.97 (1.25–3.10)**		**2.30 (1.36–3.89)**		**1.70 (1.21–2.39)**		**2.62 (1.75–3.91)**		**2.00 (1.70–2.35)**	
Income gap	Yes	67	212	19	147	0	39	8	52	67	322	51	197	47	158	35	191	294	1318
	No	117	510	66	493	69	987	59	766	100	607	104	397	286	762	168	820	969	5342
	OR 99%	1.38 (0.88–2.15)		0.97 (0.47–1.97)		not calculated		2.00 (0.72–5.54)		1.26 (0.81–1.97)		0.99 (0.60–1.62)		0.79 (0.50–1.26)		0.89 (0.53–1.51)		**1.23 (1.02–1.49**)	
Negative attitudes about sex and violence	Yes	35	75	40	268	21	218	28	315	33	169	56	109	115	229	54	104	382	1487
	No	154	664	51	387	51	816	42	545	135	775	106	501	215	676	151	914	905	5278
	OR 99%	**2.01 (1.14–3.55)**		1.13 (0.63–2.03)		1.54 (0.77–3.08)		1.15 (0.60–2.22)		1.12 (0.65–1.94)		**2.43 (1.48–3.99)**		**1.58 (1.11–2.25)**		**3.14 (1.96–5.03)**		**1.50 (1.26–1.78)**	
Feels himself to be at risk of getting AIDS	Yes	120	428	38	245	33	321	42	381	49	292	83	249	137	298	83	248	585	2462
	No	56	262	37	358	38	691	27	420	106	607	59	314	192	597	98	675	613	3924
	OR 99%	1.31 (0.83–2.08)		1.50 (0.80–2.82)		**1.87 (1.00–3.51)**		1.71 (0.89–3.30)		0.96 (0.59–1.56)		**1.77 (1.09–2.89)**		**1.43 (1.02–2.01)**		**2.31 (1.51–3.52)**		**1.52 (1.29–1.79)**	
Negative attitudes to AIDS	Yes	16	24	14	69	7	64	3	77	10	39	11	19	46	61	16	25	123	378
	No	173	715	77	595	65	970	67	783	158	905	151	591	291	863	189	1000	1171	6422
	OR 99%	**2.76 (1.20–6.31**)		1.57 (0.70–3.53)		1.63 (0.56–4.75)		0.46 (0.10–2.07)		1.47 (0.58–3.74)		**2.27 (0.85–6.04)**		**2.24 (1.33–3.77)**		**3.39 (1.51–7.57)**		**1.78 (1.35–2.35)**	

**Table 12:** Female respondents: Associations with domestic physical violence (number of responses, Odds Ratio and 99%confidence interval)

		Botswana		Lesotho		Malawi		Mozambique		Namibia		Swaziland		Zambia		Zimbabwe		Overall	
		Partner violence		Partner violence		Partner violence		Partner violence		Partner violence		Partner violence		Partner violence		Partner violence		Partner violence	

		Yes	No	Yes	No	Yes	No	Yes	No	Yes	No	Yes	No	Yes	No	Yes	No	Yes	No

Reported having multiple partners	Yes	75	171	96	267	10	54	36	227	41	102	53	103	82	77	57	113	450	1114
	No	159	755	86	599	164	1278	111	979	189	993	139	518	447	798	184	933	1479	6853
	OR 99% CI	**2.08 (1.37–3.16)**		**2.50 (1.65–3.81)**		1.44 (0.58–3.58)		1.40 (0.83–2.37)		**2.11 (1.27–3.51)**		**1.92 (1.17–3.15)**		**1.90 (1.24–2.93**		**2.56 1.62–4.03**		**1.87 (1.60–2.20)**	
Income gap	Yes	123	558	110	502	4	58	19	171	129	524	114	382	357	575	98	516	954	3286
	No	128	538	91	564	171	1340	125	997	101	614	98	412	176	390	153	701	1043	5556
	OR 99% CI	0.93 (0.65–1.33)		1.36 (0.91–2.02)		0.54 (0.14–2.04)		0.89 (0.45–1.73)		**1.50 (1.03–2.17)**		1.25 (0.84–1.87)		**1.38 (1.03–1.84)**		0.87 (0.60–1.25)		**1.55 (1.36–1.76)**	
Negative attitudes to sex and violence	Yes	32	88	87	359	42	314	45	401	38	115	48	109	165	190	30	95	487	1671
	No	224	1024	120	727	134	1093	103	824	195	1031	172	696	363	762	222	1147	1533	7304
	OR 99% CI	1.66 (0.95–2.91)		1.47 (0.99–2.19)		1.09 (0.67–1.77)		0.90 (0.55–1.46)		**1.75 (1.04–2.93)**		**1.78 (1.09–2.92)**		**1.82 (1.33–2.50)**		1.63 (0.93–2.88)		**1.39 (1.29–1.79)**	
Feels herself to be at risk of AIDS	Yes	168	664	104	454	84	486	89	586	110	472	131	380	214	322	124	399	1024	3763
	No	66	385	63	530	89	874	51	563	103	591	68	348	298	614	95	696	833	4601
	OR 99% CI	1.48 (0.98–2.22)		**1.93 (1.24–2.99)**		**1.70 (1.12–2.57)**		**1.68 (1.04–2.69)**		1.34 (0.91–1.97)		**1.76 (1.15–2.70)**		**1.37 (1.02–1.83)**		**2.28 (1.56–3.33)**		**1.50 (1.32–1.72)**	
Negative attitudes to AIDS	Yes	8	15	13	84	14	96	10	86	6	21	15	14	46	39	6	28	118	383
	No	249	1098	194	1012	162	1314	138	1139	227	1127	206	799	492	932	246	1218	1914	8639
	OR 99% CI	2.35 (0.77–7.14)		0.81 (0.37–1.78)		1.18 (0.55–2.55)		0.96 (0.39–2.34)		1.42 (0.43–4.72)		**4.16 (1.68–10.30)**		**2.23 (1.27–3.94)**		**1.06 (0.33–3.43)**		**1.39 (1.05–1.84)**	

### Multiple partners

One in every four respondents (4,468/17,948) who answered the question  reported having two or more sexual partners in the last year; 15.9%  (3,276/20,639) declined to answer. The proportion reporting multiple  partners, out of those who had partners in the last year, varied somewhat  by country: Botswana 32.1% (566/1,760), Lesotho 43.9% (780/1,760), Malawi  12.5% (274/2,195), Mozambique 31.6% (706/2,212), Namibia 21.0% (440/2,062),  Swaziland 35.1% (517/1,465), Zambia 26.0% (600/2,316) and Zimbabwe 26.8%  (585/2,175).

Using two or more partners in the last 12 months as a definition of multiple partners, there was a strong association with partner physical violence: female respondents OR 1.87 99%CI 1.46–2.41 (450/1564 of those with two or more partners compared with 1479/8332 among those with one on no partners) and male respondents OR 2.00 99%CI 1.47–2.66 (627/2755 among those with two or more partners compared with 592/4616 among those with one or no partners).

In all age groups in all countries, having multiple partners was a risk factor for violent altercations. A logistic model taking into account country, food security, sex of respondent, income, education and employment accentuated the risk of violent altercations for people with multiple partners (unadjusted OR 1.75, adjusted OR 2.03 99%CI 1.65–2.42, indicating underestimation of the unadjusted estimate).

Partner physical violence increased progressively with number of partners in the last 12 months: 234/1689 (13.9%) with no partners, 16.3% (1849/11324) with one partner, 22.7% (516/2269) with two partners, 25.4% (253/1034) with three partners, 29.2% (118/405) with four and 29.2% (185/633) with five or more partners reported domestic physical violence in the last year (χ^2 ^199.8, 5 df).

### Community dynamics and collective efficacy

A large proportion of the sample (65%, 12760/19626) said that domestic violence was considered a serious issue in their community (4.9% missing data, 1004/20639). Yet two thirds (9944/15880) of those who did not report physical violence and one half of those reporting partner physical violence in the last year (1654/3336) had never spoken about it. Those who spoke about it did so most frequently with friends (50.0% 3754/7504) and family (24.2%, 1819/7504). One in every ten said they had discussed with a neighbour (720/7504) and another one in ten with a partner or spouse (745/7504). There were no remarkable differences between male and female respondents, or between those who reported violent altercations and those who had not done so.

Over one half of the respondents said that their community could do something about violence against women (unweighted 56.2% based on 10466/18617, missing data 2017/20639 or 9.7%). Male respondents were more likely to express collective efficacy (OR 1.12 99%CI 1.02–1.23, 4529/7828 male and 5879/10685 female respondents felt their communities could do something about violence against women). Collective efficacy was highest in Botswana (75.6% 1715/2268) and Lesotho (62%, 1299/2095) and lowest in Zambia (44.5%, 1215/2732).

### Relevance of partner physical violence to HIV risk

People who reported partner physical violence (male or female) were significantly more likely to believe they were at risk of getting HIV (OR 1.51, 99%CI 1.37–1.68; 1615/3075 who reported partner physical violence and 6261/14832 who did not report partner physical violence said they were at risk of HIV infection). This was not explained by country, sex of the respondent or any of the factors we could test in this study.

The average male respondent who reported partner physical violence was significantly more likely to anticipate a negative reaction to knowing he was HIV positive (no change, spread intentionally, sleep with virgin, etc) compared with one who had not suffered violence in the last year (OR 1.51, 99%CI 1.23–1.83, 286/1163 among those reporting and 1089/6142 not reporting partner physical violence). This association did not hold for female respondents, and among men it was not explained by country or any of the other variables we could test (List 1, see appendix).

## Discussion

High rates of domestic physical violence in all eight countries were conspicuously independent of education, household size, household income and remunerated employment. After taking into account age, sex, country and other factors, domestic physical violence was strongly associated with income gradients (being unemployed in the context of some household income) and home language in one country, and with multiple partners in the last year in all countries. Victims of partner physical violence were more likely to feel at risk of HIV infection and more likely to anticipate antisocial behaviour if they found they were HIV positive.

This is a cross-sectional household survey based on face-to-face interviews. This design limits conclusions about causality of, for example, multiple partners leading to physical violence or being the consequence of physical violence. It is likely that some respondents held back from expressing their true belief or experience. Even with the best field practices – including independent translation and back-translation of questionnaires, standardised training of local interviewers, in-country piloting and consultation with local community representatives, double-data entry and verification – measurement error is possible. The sample makes the results relevant to the eight countries, but not necessarily to other countries.

A major limitation is that we only considered domestic *physical *violence. This almost certainly underestimates the level of domestic violence. Other forms (verbal, sexual, economic and psychological) were beyond the scope of the study. In all countries we asked the same questions of men and women. We were able to examine several intermediate outcomes related to domestic violence – including attitudes, subjective norms, collective efficacy and discussion/socialisation – but most of these could be addressed only superficially through one or two items in the questionnaire.

We had no measure of *severity *or frequency of physical domestic violence, making it difficult to interpret the proportion of men and women who reported partner violence in the last year. Large studies in the UK and USA have reported similar proportions of partner violence for males and females, but found male on female violence to be more severe than female on male violence[23,24]. It is quite possible that the same is true for southern Africa. The men we interviewed were at home during working hours and, in this respect at least, they may not be typical of all men in the eight countries. We also did not ask who initiated the altercation, so it is also possible these reports reflect women defending themselves from male-initiated violence. Even so, the finding is compatible with a degree of female agency in domestic physical violence and supports our conclusions from South Africa that initiatives against sexual violence should look beyond gender stereotypes of victims and villains[25].

There was no recognisable pattern of poverty and domestic violence between countries (Mozambique, the poorest country, reported the lowest rates while Zambia reported the highest). We also did not find significant associations between victims and their individual education or employment, and we could only address the income gradient between partners through a proxy variable. It is possible that in-household inequality in education and income could be more relevant to domestic violence than we were able to measure in this study[26]. There was no interpretable association between the Gini coefficient (measuring inequality in the country) and male or female reports of violence (Tables 3 and 4). The Gini coefficient used for Botswana and Lesotho was 0.63, Malawi 0.50, Mozambique 0.40, Namibia 0.74, Swaziland 0.61, Zambia 0.42 and Zimbabwe 0.61[27].

The occurrence of domestic physical violence in some parts of Zambia raises the question of something being done differently there, despite efforts to reproduce exactly the same survey in all countries. Whatever the reason for the higher rates of domestic physical violence detected in Zambia, it seems unlikely the same error lies behind the inability to demonstrate an association between violent altercations and education, overcrowding, income and age – consistent across all the countries.

## Conclusion

If there is good news from this study, it is that multiple partners, attitudes and subjective norms are more in the control of most individuals than are poverty, overcrowding and education – without detracting from the need for massive investment in these sectors.

An unanswered question is how to modify attitudes or multiple partners. There is also no guarantee that changing attitudes will, on its own, impact on behaviour. The study confirms the importance of moving beyond gender stereotypes of victims and villains. Men also report suffering partner physical violence, although our inability to measure severity could mask an important gender difference. The solutions to domestic violence lie with both men and women, and both have agency in this regard. There was also a prominent sense of collective efficacy, the majority expressing they could do something about domestic violence.

Although many thought their community could deal with violence against women, few victims and still fewer of the non-victims said they had discussed violence against women with anyone. Stimulating discussions about violence against women offers one direction for initiatives against partner physical violence. Wider discussion could influence social norms, in addition to targeting individual attitudes and supportive public policy.

## Appendix

List 1. Variables tested sequentially, from which independent associations were included in logistic regression model

### Individual and household characteristics

How many people live in the household

Age and sex of each one

Language spoken at home most of the time

Last grade of education respondent completed

Main occupation of respondent

Total household income per month

Did household have enough food in the last week

Was the respondent alone or was someone listening

### HIV risk

Do you think you are at risk of getting HIV

If you found you were HIV positive, how would you change your sex life

### Sexual violence

If a woman gets raped its her own fault.

Forcing sex with someone you know is not rape.

Forcing your partner to have sex is rape.

### Subjective norms about sexual violence

Do most people in your community feel forcing your partner to have sex is rape?

Do most people in your community feel women have a right to refuse sex with their partners?

Is violence against women considered a serious problem in this community?

### Collective efficacy about sexual violence

Can your community do anything about violence against women?

### Attitudes to domestic violence

Women have the right to refuse to have sex with partner

Violence between a man and a woman is a private matter Women sometimes deserve to be beaten.

### Subjective norms about domestic violence

Do most people in your community feel women sometimes deserve to be beaten?

### Discussion about domestic violence

In the last year, how often did you talk with anyone about domestic violence? [never, seldom or often]

To whom did you speak most often?

### Practices relating to domestic violence

What community activity about violence against women have you participated in?

In the last year, have you and your partner had violent arguments where someone was physically hurt?

### Transactional sex

Men have the right to have sex with their girlfriends if they buy them gifts.

Its okay for an older man to have sex with teenagers

A person has to have sex with their boyfriend or girlfriend to show that they love them.

Do most of your friends feel men have the right to sex with their girlfriends if they buy them gifts?

## Competing interests

All authors declare that there is no competing interest. Esca Scheepers and Sue Goldstein were employed by Soul City,  which subcontracted the national education-entertainment programmes in the  eight countries.

## Authors' contributions

NA was involved in study and questionnaire design, statistical analysis, drafting manuscript. AHF was involved in statistical analysis, interpretation and drafting manuscript. SM was involved in study design, acquisition of data, drafting manuscript. ES and SG were involved in study and questionnaire design, analysis and interpretation, drafting manuscript, administration and technical support. NA, AHF, ES and SG had full access to all data and take responsibility for the integrity of the data and accuracy of data analysis. All authors read and approved the final manuscript.

## Pre-publication history

The pre-publication history for this paper can be accessed here:


